# Comparative Genomics Reveals Ancient and Unique Pathogenicity Features in Australian *Fusarium oxysporum* f. sp. *vasinfectum*

**DOI:** 10.3390/jof11070481

**Published:** 2025-06-25

**Authors:** Angel David Popa-Baez, Linda J. Smith, Warwick N. Stiller, Melanie Soliveres, Gunjan Pandey, Christopher A. Saski, Don C. Jones, Iain W. Wilson

**Affiliations:** 1 Agriculture and Food, Commonwealth Scientific and Industrial Research Organisation (CSIRO), Canberra, ACT 2601, Australia; 2 Ecosciences Precinct, Department of Primary Industries, Dutton Park, QLD 4102, Australia; 3 Agriculture and Food, Commonwealth Scientific and Industrial Research Organisation (CSIRO), Narrabri, NSW 2390, Australia; 4 Environment, Commonwealth Scientific and Industrial Research Organisation (CSIRO), Canberra, ACT 2601, Australia; 5 Department of Plant and Environmental Sciences, Clemson University, Clemson, SC 29631, USA; 6 Cotton Incorporated, Cary, NC 27513, USA

**Keywords:** Fusarium wilt, *Fusarium oxysporum* f. sp. *vasinfectum*, comparative genomics, chromosome-level assembly, australian FOV isolates, pathogen evolution

## Abstract

*Fusarium oxysporum* f. sp. *vasinfectum* (*Fov*) is a devastating cotton pathogen. Australian *Fov* strains are distinguished by their ability to infect plants without nematode interaction and are genetically distinct from global *Fov*, classified into two vegetative compatibility groups (VCG-01111 and VCG-01112). Here, we present chromosome-level genome assemblies of a historical isolate for each Australian *Fov* VCG. The end-to-end gapless genome assemblies demonstrate high contiguity and completeness, with 97.7% BUSCO completeness for both isolates. Phylogenetic analysis indicates that the Australian *Fov* lineages diverged from other known *Fov* genomes over 3.6 million years ago, while VCG-01111 and VCG-01112 separated approximately 1.1 million years ago. Comparative genomics analysis identified four accessory chromosomes unique to the Australian isolates. Functional annotations revealed 14,495 and 15,342 genes in VCG-01111 and VCG-01112, respectively, with accessory chromosomes containing significantly fewer genes than core chromosomes. Ortholog analysis uncovered unique gene clusters enriched in key metabolic pathways, pathogenicity, and cell division processes. Additionally, we identified several novel lineage-specific peptides unique to each Australian isolate. This comprehensive genomic characterization provides the first insights into the unique evolutionary history of Australian *Fov*, distinguishing them from global *Fov* races. Our findings lay the foundation for understanding the genetic factors underlying their exceptional virulence, which makes Australian *Fov* among the most aggressive cotton pathogens worldwide.

## 1. Introduction

Fusarium wilt (FW) of cotton is caused by the soil-borne fungal pathogen *Fusarium oxysporum* f. sp. *vasinfectum* (*Fov*). The fungus infects cotton roots and colonizes the vascular system, where it proliferates and induces vessel occlusion resulting in plant wilting, stunting, defoliation and death [1,2]. The pathogen’s ability to persist in soil for decades as chlamydospores poses a significant challenge for disease management, making the development of resistant cotton cultivars the primary strategy for combating the disease [3,4,5]. Classical and molecular characterization of *Fov* has led to the identification of multiple races globally [6], (race 1–7), which are classified into 23 recognized and several undetermined vegetative compatibility groups (VCGs) [7]. These VCGs are thought to represent clonal lineages [4,5] with VCG-0111 to VCG-01110 encompassing strains pathogenic to cotton [5]. In culture, Australian *Fov* strains are morphologically identical to each other but are distinguished by the production of unique pigmentation, and a characteristic volatile odour not observed for other *Fov* isolates. Notably, these strains are highly pathogenic in the absence of nematode infection [2]. However, Australian isolates of *Fov* are similar to race 6 on standard differential host assays. Consequently, they have been assigned to distinct vegetive compatibility groups VCGs 01111 and 01112 [2]. Molecular analyses using gene sequences and markers have confirmed the genetic distinctiveness of Australian *Fov* relative to the global *Fov* population. These studies have established that the Australian *Fov* is closely related to a lineage of indigenous *F. oxysporum* found in several areas of Australia, including the Darling Downs in Queensland [7,8,9].

Australia is Indigenous to 17 wild *Gossypium* species, 4 (*G. australe, G. bickii, G. nelsonii* and *G. sturtianum*) in which host ranges are known to overlap or abut areas where the majority of cultivated cotton is grown [10]. A survey of *Fusarium* spp. associated with these wild cottons identified a number of *F. oxysporum* isolates that caused mild but typical FW symptoms on cultivated cotton [9], suggesting that two *Fov* VCGs found in commercial cotton fields evolved from local populations that were associated with native cotton species.

Fusarium wilt (FW) was first identified in Australia in 1993 [11] and spread rapidly, emerging as one of the most economically destructive diseases affecting Australian cotton production. The widespread susceptibility of commercial cultivars resulted in production losses of virtually 100% in some fields [12]. Although the inheritance of host resistance to FW is complex, significant progress has been made in developing cultivars with genetic resistance [13]; however, the disease remains of significant economic concern. The two Australian *Fov* VCGs have different geographic distributions with VCG 01111 occurring in all infected cotton-growing regions, while VCG 01112 is restricted to the Boggabilla region of northern NSW where it was first detected. However, cultivar resistance based on vascular browning scores does not appear to be significantly different between the two VCGs [14].

Understanding the evolutionary origin and relationships of pathogens is important for effective disease management. The unique evolutionary trajectory and independent origin of Australian isolates raises important questions about their genomic adaptations and virulence mechanisms., which can be revealed through extensive comparative genomics studies. For instance, gene family evolution and metabolic adaptations in plant pathogens often reflect their environmental pressures and host specialization [15,16]. Understanding these changes can reveal mechanisms of host colonization and tissue specificity, which can help understand deviations and the key characteristics that identify an Australian biotype. For instance, in *F. oxysporum* f. sp. *lycopersici* (*Fol*)*,* the causal agent of tomato wilt, different races present distinct behaviours *in planta,* which can help the pathogen overcome the plant resistance mechanism [17]. In *Fol*, the genome is divided into two groups: core chromosomes and accessory chromosomes. This latter group primarily hosts the pathogenic Secreted in Xylem genes (SIX) [18], highlighting the importance of accessory chromosomes for the pathogenicity of *F. oxysporum* pathogens [19].

In the case of *Fov*, several studies have tried to identify which SIX proteins are shared by the Australian biotypes, which have shown that SIX6 appears to be present in both Australian biotypes but not in other races or VCGs of *Fov.* The limited number of high-quality genomes for the *Fov* races complicates the study of these homologues [20,21,22]. Although recently, several complete genomes of some *Fov* races have become available. For instance, *Fov* race 7, like in *Fol*, also contained accessory chromosomes, which also carried ortholog SIX genes like *Fol* and other *Fov* race 7 specific genes [20]. Comparative genomic and functional validation studies have shown that these SIX proteins are crucial factors that contribute to the pathogenicity of *Fov* race 7 [20]. Therefore, studying the evolution of these genomic elements can provide insights into host adaptation and virulence mechanisms.

Thus, given the uniqueness and potential origin of new Australian isolates, in this study, we have built two new genome assemblies for the Australian biotypes VCG-01111 and VCG-01112 to aid researchers in identifying genetic differences between *Fov* races and potentially uncovering mechanisms capable of increasing cotton resistance to these devastating diseases. Using these new complete genomes, we conducted comparative genomic analyses to characterize their unique features. We focused on genome architecture, gene family evolution and pathogenicity determinants, including SIX proteins and novel secreted peptides. This analysis provides insights into the genomic differences between these isolates and other *Fov* strains, revealing potential avenues for understanding their enhanced virulence and regional adaptation.

## 2. Materials and Methods

### 2.1. Fungi Isolate Collection

Two *Fov* isolates were obtained from the Department of Agriculture and Fisheries (now the Department of Primary Industries), Queensland (QLD), Australia culture collection. These isolates were initially recovered from cotton, displaying typical symptoms of Fusarium wilt, grown in fields in Queensland (QLD) and New South Wales (NSW), Australia, during the early to mid-1990s. Specifically, isolate ATCC24233 (VCG-01111) was collected from the Cecil Plains in the Darling Downs region of Queensland (QLD) in May 1993, while isolate ATCC24673 (VCG-01112) was isolated from a property in Boggabilla, MacIntyre Valley, New South Wales (NSW) in January 1995. The latter strain was isolated from the Siokra 1–4 cotton variety.

### 2.2. DNA Extraction and Quality Assessment

The *Fov* isolates were maintained in 15% glycerol at minus 80 °C and recovered on potato dextrose agar (¼ PDA) [23] at 25 °C for 4–7 days. Mycelium was aseptically scraped from the plate, snapped, frozen in liquid Nitrogen and stored at minus 80 °C. Genomic DNA was extracted by The Westmead Institute for Medical Research, using a modified CTAB method [24]. First, 300 mg of dried mycelium powder was mixed with 10 mL CTAB buffer (2% CTAB, 100 mM Tris-HCl pH 8.0, 20 mM EDTA pH 8.0, 1.4 M NaCl) and incubated at 65 °C for 30 min. Then, the lysate was treated with 50 µg/mL RNase A and 3 M sodium acetate (pH 5.2) at 37 °C for 30 min. DNA was purified using sequential extractions with phenol–chloroform–isoamyl alcohol (25:24:1) and chloroform–isoamyl alcohol (24:1), followed by precipitation with an equal volume of isopropanol at −20 °C for 2 h. The pellet was washed with 70% and 100% ethanol, air-dried and dissolved in 0.1 mL TE buffer. The extracted DNA was stored at −80 °C until being used for HiFi long-read sequencing in a PacBio Revio at the AGRF, Sydney, Australia.

Snap-frozen hyphae from both isolates were submitted for library preparation using a Phase Genomics Proximo Hi-C Fungal kit (Phase Genomics, Seattle, WA, USA) and sequenced on the Illumina NovaSeq X Series (Illumina Inc., San Diego, CA, USA). This work was performed by the Biomolecular Resource Facility—an NCRIS-supported Bioplatforms Australia genomics node at The John Curtin School of Medical Research, ANU.

### 2.3. Genome Assembly and QC

The genome size of both strains was estimated using the default Genomescope2 (v2.0.1) parameters with 19 k-mers [25]. The short (HiC) and long reads (HiFi) were filtered to 60X of the estimated genome size and assembled with default Mabs (hifiasm) under default conditions [26,27]. The resulting assembly was decontaminated with NCBI’s FCS tool (v0.5.4) [28] and scaffolded into chromosomes with the default YAHS pipeline (v1.2.1) [29]. The HiC maps were manually curated in Juicebox (v2.20.00) [30] to generate the final genome assembly. Genome assembly quality was assessed using BlobToolKit (v4.2.1), which reviewed the genome assemblies’ statistics to produce a Snail plot for each genome. Additionally, the Benchmarking Universal Single-Copy Orthologue (BUSCO) (v5.4.7) score was calculated for each genome, as well as its genome composition. We also verified the location of telomeric regions using quarTeT, a telomere-to-telomere toolkit to identify gaps, centromere and telomeres in genome assemblies [31].

### 2.4. Genome Annotation

Genome annotation was conducted using the funannotate pipeline (v1.9.1) [32]. First, we removed repetitive contigs with funannotate clean and soft-masked repetitive elements in the remaining scaffolds. Following this, we predicted gene sequences using “fusarium” as a BUSCO seed [33]. Functional annotation of the predicted genes was performed using interProScan (v5.73-104.0) [34] and antiSMASH (v 7.0) [35] to identify protein families and secondary metabolites.

We used SignalP (version 6) [36] to identify signal peptides using the “Eukarya” as the organism, fast model and short output instructions. SignalP utilises protein language models, which generate a semantic representation of proteins to identify biological and structural properties from the protein sequence. This determines the N-terminus of proteins, directing them to secretory pathways, and assigns a probability score to each protein as a signal peptide. We also identified which of the annotated proteins are also classified as transmembrane domain proteins using DeepTMHMM [37] and MULocDeep [38]. This additional validation limits the false positive rate since true signal peptides can be predicted as signal peptides by SignalP and not as transmembrane domains by DeepTMHMM and MucLoc.

### 2.5. Comparative Genomics and Gene Orthology Analysis

Gene Orthology (GO) and phylogenetic analysis of the assembled and annotated genomes were performed using the OrthoVenn3 comparative genomics platform [39]. For this analysis, we selected only the eight chromosome-level *F. oxysporum* genomes (Table S1), with *Fol* and *F. verticillioides* (*Fve*) serving as outgroups. For the orthologous analysis with OrthoVenn3 (v3), we used the OrthoFinder (v3.0.1) [40] algorithm with an E-value filter of 0.001 and an inflation value of 1.50. The phylogenetic analysis model was JTT+CAT. Gene family expansion and contraction were estimated with a divergence time of 5.34 million years ago (MYA) for *F. oxysporum* and *F. verticillioides*, which were obtained from TimeTree5 [41]. This value was derived from four different studies [42,43,44,45]. Finally, we identified a lineage-specific scaffold for the two new genomes by comparing sequence alignments with the *Fol* and *Fve* genome assemblies using D-GENIES (v1.5.0) [46].

## 3. Results

### 3.1. Genome Assembly

**Figure 1:**
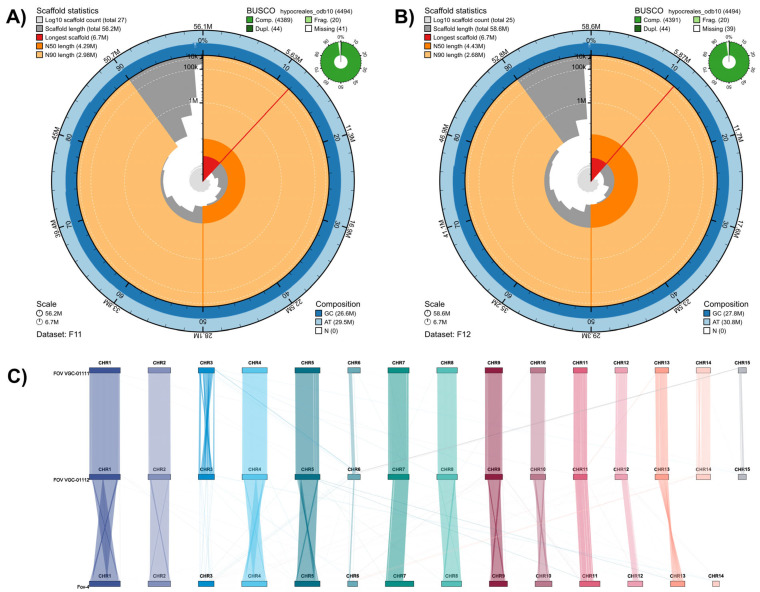
Genome assembly statistics for the two Australian biotypes. (**A**) Snail plot for the VCG 01111 genome assembly showing the BUSCO and genome contiguity with description of the N50 and N80 length per scaffold, as well as the GC, AT and N-gaps content. (**B**) Snail plot showing similar genome statistics for the VCG-01112 genome assembly. (**C**) Pairwise genome synteny for both Australian biotypes in relation to *Fusarium oxysporum* f. sp. *lycopersicum*.

We generated two highly contiguous and complete de novo genomes for Australian *Fusarium oxysporum* f. sp. *vasinfectum* isolates, VCG-01111 and VCG-01112. The new assemblies contain all 15 chromosomes and showed similar statistics: N50 scaffold length of 4.3 Mb and 4.43 Mb, 47.3% and 47.5% GC content, no gaps and BUSCO completeness of 97.7% for both FOV VCG-01111 and FOV VCG-01112, respectively (Figure 1). These statistics highlight the contiguity and completeness of the two de novo genome assemblies obtained using our hybrid assembly approach, which combines HiC and HiFi reads (Figures S1 and S2). In addition, this approach has allowed us to generate two end-to-end genome assembly instances, with the VCG-01112 genome assembly containing the telomeric regions in 10 of the 15 chromosomes (Figure S3). This finding could also explain the minor genomic size differences between the two assemblies (Figure 1).

Pairwise alignment of both assemblies with *F. oxysporum* f. sp. *lycopersici* (*Fol*), FOV race 1, FOV race 4 and FOV race 7 were used to reorient the scaffolds and identify the core and accessory chromosomes (Figures S4 and S5). We identified 11 chromosomes that shared a high level of synteny with the *Fol* chromosomes; thus, we designated them as core chromosomes, and the remaining four (CHR3, CHR6, CHR14 and CHR15) were characterised as accessory chromosomes (Figure 1).

Three of the accessory chromosomes were not syntenic. First, CHR3 showed a high level of rearrangements within the chromosome, with only the ends of the chromosomes showing synteny between the genomes and a small group of chromosome segments aligning with other core and accessory chromosomes (Figure 1C). Second, only partial sections of CHR6 and CHR15 showed collinearity or alignment with other chromosomes, indicating potential regions private to each isolate (Figure 1C).

### 3.2. Genome Annotation and Functional Characteristics

Functional annotation for the VCG-01111 genome identified 14,495 genes, with a total size of 22,333 kb and a mean size of 1483 bp, which accounted for 39.57% of the genome. For the VCG-01112, the gene total number was 15,342 with a total size of 22,609 and a mean average of 1478 bp, which accounted for 38.75% of the genome. We also observed a smaller number of genes in some of the accessory chromosomes (CHR3, CHR6, CHR14 and CHR15), with only an average of 23% of the VCG-01111 accessory chromosomes containing coding genes and 30% of VCG-01112 accessory chromosomes containing the gene coding sequence. However, this difference was not significantly different from the core chromosomes as some also had low gene content (Figure S6).

Ortholog analysis for the eight *Fov* genomes revealed 19,414 ortholog clusters, with 6962 single-copy clusters; this came directly out of the 132,987 proteins present across eight *Fov* genomes, 5268 (3.96%) of which were singletons. In the case of the Australian genomes, we found 121 singletons for VCG-01111 and 132 for VCG-01112; however, the cluster for both genomes had 442 clusters of orthologs. VCG-01112 had 11 unique gene clusters with 29 genes, whose Gene Ontology enrichment results showed that the most significant functional specialisation was DNA template and transcription (two genes, *p* < 0.001); this was accompanied by enrichment in the lipid metabolic process (four genes, *p* < 0.001). In contrast, VCG-01111 only had three gene clusters with seven genes unique to this isolate, which were not enriched.

When we looked at the 442 gene clusters for the Australian genome clusters, Gene Ontology enrichment analysis revealed significant functional specialization across biological processes, molecular functions and cellular components. The most significantly enriched biological process was DNA-templated transcription (21 genes, *p* < 0.0001), accompanied by multiple regulatory mechanisms, including both positive and negative regulation of RNA polymerase II-mediated transcription (*p* < 0.0001) (Table S2).

Notably, pathogenesis-related processes showed substantial enrichment (nine genes, *p* < 0.0001), indicating potential host–interaction capabilities. This was complemented by enrichment in protein transport (four genes, *p* < 0.0001) and sporulation processes (four genes, *p* < 0.0001). Carbohydrate metabolism emerged as a functional category, significantly enriching catabolic and transport processes. Specifically, we observed enrichment in carbohydrate transport (six genes, *p* < 0.0001) and various polysaccharide degradation pathways, including cellulose (four genes, *p* < 0.0001), xylan (four genes, *p* < 0.0001) and pectin (six genes, *p* < 0.0001) catabolism (Table S2).

Additional metabolic capabilities were indicated by enrichment in lipid metabolism (*p* < 0.0001), protein folding (*p* < 0.0001) and various biosynthetic processes, including sterol (*p* < 0.0001) and GPI anchor biosynthesis (*p* < 0.0001). The presence of multiple transmembrane transport processes, including drug transport (*p* < 0.0001) and amino acid transport (*p* < 2.17 × 10^−16^), suggests sophisticated metabolite trafficking systems (Table S2).

At the cellular component level, significant enrichment was observed in integral membrane components (five genes, *p* < 0.0001) and nuclear localization (three genes, *p* < 0.0001), consistent with the observed enrichment in transcription and transport processes. The molecular function analysis revealed a pronounced enrichment in oxidoreductase activity (22 genes, *p* < 0.0001) and zinc ion binding (9 genes, *p* < 0.0001), along with diverse enzymatic activities including transferases (5 genes, *p* < 0.0001), monooxygenases (6 genes, *p* < 0.0001) and protein kinases (4 genes, *p* < 0.0001) (Table S2).

### 3.3. Evolutionary Differences and Gene Family Evolution

**Figure 2:**
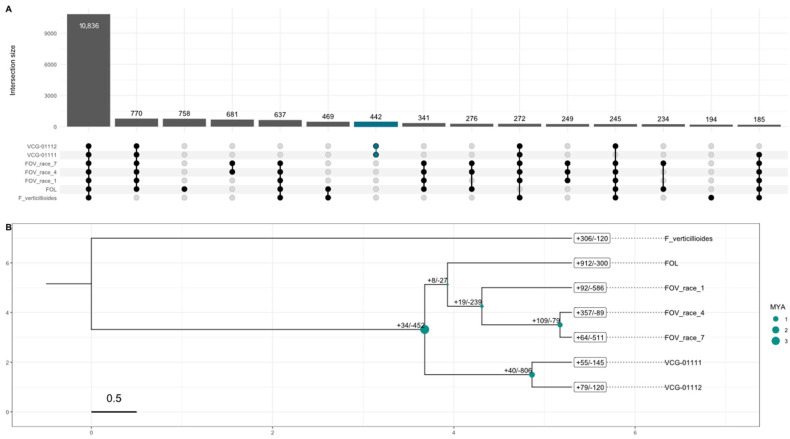
Gene orthology (GO) analysis results and gene family analysis with published *Fov* genomes. (**A**) Upset plot for the Gene Orthology clusters found via Orthovenn3 showing the unique gene clusters for different genomes and genome pairs. (**B**) Phylogenetic tree generated with CAFE5 showing the time-calibrated phylogenetic relationship between *Fov* genomes, as well as the gene family gain and losses over evolutionary time; branch length shows times in 0.5 MYA interval calibrated based on the estimated time split between *F. verticillioides* and *F. oxysporum* of 5.3 MYA.

Gene family evolution analysis with CAFE revealed dynamic patterns of gene family expansions and contractions across the phylogenetic timeline spanning approximately 5.3 million years since the separation of *F. oxysporum* and *F. verticillioides*. The analysis identified distinct evolutionary patterns across the different lineages (Figure 2B). The outgroup species *F. verticillioides* showed moderate gene family evolution with 306 expansions and 120 contractions, providing context for this group’s ancestral state of gene families (Figure 2A). However, an interesting result is the relationship between the Australian biotypes and the other *Fov,* in which *Fol,* which had the most dramatic gene family expansion (Figure 2B), is more closely related to all the *Fov* genomes in our study than the two Australian biotypes. In fact, the two Australian biotypes most common recent ancestor with *Fol* and the other *Fov* occurred around ~3.67 MYA preceding the split of *Fol* and the other *Fov* genomes’ most recent common ancestor. Interestingly, the most recent split in our time-calibrated gene family tree is between Race 4 and Race 7, which occurred around 0.86 MYA. These two races contained the largest gene expansion (+109) of any other node splits. The significant increase in gene family size and the unique set of gene clusters linked to these two races could explain some of their unique pathogenic characteristics, especially in the case of Race 4, which does not need cooperation with nematodes to infect cotton plants. Furthermore, in the same clade, we observed that Race 1 occurred ~371,000 years after the split from *Fol* which our phylogenetic analysis estimate occurred ~250,000 years after the most recent common ancestor that preceded the evolution of the two Australian biotypes.

The split between VCG 01111 and VCG 01112 is of particular interest as it revealed contrasting patterns of gene family evolution. VCG-01112 exhibited 79 expanded and 120 contracted gene families, while VCG-01111 showed 55 expansions and 145 contractions (Figure 2B). Their immediate common ancestor underwent substantial gene family changes, with 40 expansions and 806 contractions, suggesting a period of significant genome refinement (Figure 2B).

Functional enrichment analysis of gene families in VCG-01111 revealed a distinctive pattern of adaptation, particularly in stress response and metabolic processes (Table S3). The most significantly enriched biological process was the cellular response to xenobiotic stimulus (31 genes, *p* < 0.0001), complemented by the response to antibiotic (19 genes, *p* < 0.0001), and other molecular functions involved in biosynthesis and biodegradation of compounds, which suggest sophisticated xenobiotic response mechanisms.

Metabolic functions showed significant specialization, with oxidoreductase activity being highly enriched (9 genes, *p* < 0.0001). Notable metabolic adaptations included polyamine catabolism (18 genes, *p* < 0.0001), styrene metabolism (11 genes, *p* < 0.0001), and alcohol metabolic processes (9 genes, *p* < 0.0001). More specific catabolic processes were also enriched, including tyrosine catabolism (10 genes, *p* < 0.0001), arginine catabolism (4 genes, *p* < 0.0001) and ethanol catabolism (5 genes, *p* < 0.0001) (Table S3).

Transcriptional regulation and chromatin modification emerged as important functional categories, with significant enrichment in DNA-templated transcription (15 genes, *p* < 0.0001) and histone acetyltransferase activity (15 genes, *p* < 0.0001). UV-damage excision repair mechanisms (17 genes, *p* < 0.0001) suggest enhanced DNA maintenance capabilities (Table S3).

Transport processes showed substantial enrichment, particularly in phosphate ion transmembrane transport (11 genes, *p* < 0.0001) and pantothenate transmembrane transport (11 genes, *p* < 0.0001). Additional transport functions included plasma membrane acetate transport (7 genes, *p* < 0.0001) and riboflavin transport (5 genes, *p* < 0.0001), supported by general transmembrane transporter activity (12 genes, *p* < 0.0001) (Table S3).

In the case of VCG-01112, functional enrichment analysis of gene families revealed distinct patterns of functional specialization, particularly in metabolic activities and regulatory processes (Table S4). The most significantly enriched molecular function was oxidoreductase activity (9 genes, *p* < 0.0001), complemented by specialized oxidoreductase activities acting on paired donors with oxygen incorporation (38 genes, *p* < 0.0001), suggesting enhanced oxidative metabolism capabilities.

A striking enrichment was observed in nucleotide-binding functions, particularly GTP binding (51 genes, *p* < 0.0001) and GTPase activity (10 genes, *p* < 0.0001), indicating expanded signal transduction and molecular switching capabilities. This was further supported by enrichment in signal transduction pathways (four genes, *p* = 0.014) and regulatory processes (Table S4).

DNA-related processes showed significant enrichment, particularly in UV-damage excision repair (17 genes, *p* < 0.0001), suggesting enhanced DNA maintenance capabilities. The enrichment in histone acetyltransferase activity (15 genes, *p* < 0.0001) and sequence-specific DNA binding transcription factor activity (20 genes, *p* < 0.0001) indicates sophisticated epigenetic and transcriptional regulation mechanisms. This was further evidenced by enrichment in negative regulation of helicase activity (10 genes, *p* < 0.0001) and positive regulation of transcription from RNA polymerase II promoter (3 genes, *p* = 0.014) (Table S4).

Pathogenesis-related functions maintained significant representation (11 genes, *p* < 0.0001), along with hyphal growth (5 genes, *p* = 0.0015), suggesting the retention of key virulence-associated capabilities. The SOS response pathway (five genes, *p* < 0.0001) indicates enhanced stress response mechanisms (Table S4).

Several metabolic processes showed significant enrichment, including methanol metabolism (10 genes, *p* < 0.0001), sulphur compound metabolism (6 genes, *p* < 0.0001) and terpenoid biosynthesis (2 genes, *p* = 0.0012). Transport-related processes were also enriched, including riboflavin transport (five genes, *p* < 0.0001) and plasma membrane acetate transport (seven genes, *p* < 0.0001) (Table S4).

Furthermore, the gene family evolution in the common ancestor of VCG-01111 and VCG-01112 revealed significant enrichment patterns associated with both expanded (+40) and contracted (−806) gene families. These families showed substantial enrichment in several key functional categories, particularly those related to stress response and regulatory processes.

Among the most significantly enriched biological processes in the expanded families were stress granule assembly (34 genes, *p* < 0.0001) and transcription elongation from RNA polymerase II promoter (66 genes, *p* < 0.0001), suggesting enhanced capability for transcriptional regulation and stress response. This was further supported by enrichment in the regulation of transcription from RNA polymerase II promoter (28 genes, *p* < 0.0001) and the regulation of mRNA stability in response to oxidative stress (20 genes, *p* < 0.0001) (Table S5).

Notably, pathogenesis-related functions were significantly enriched (20 genes, *p* < 0.0001), along with response to pheromone (24 genes, *p* < 0.0001), indicating the retention and expansion of virulence-associated capabilities, which we have already observed in FOV VCG-01112. The expansion also encompassed genes involved in key metabolic processes, including the terpenoid biosynthetic process (*p* < 0.0001) and the phospholipid metabolic process (35 genes, *p* < 0.0001) (Table S5).

Regarding molecular functions, the expanded gene families showed significant enrichment in oxidoreductase activity (24 genes, *p* < 0.0001) and metal ion binding (44 genes, *p* < 0.0001). Protein modification capabilities were also enhanced by enrichment in cAMP-dependent protein kinase regulator activity (20 genes, *p* < 0.0001) (Table S5).

Signal transduction pathways showed notable modifications, significantly enriching TORC2 signalling (28 genes, *p* < 0.0001), suggesting the refinement of cellular signalling networks, potentially affecting growth regulation and stress responses (Table S5).

### 3.4. Identification of Secreted in Xylem Proteins (SIX)

In addition to identifying gene family evolution, we looked at the SIX proteins from FOL to identify their orthologs in the two Australian genome isolates. BLAST analysis revealed the presence of several SIX gene orthologs in both FOV VCG-01111 and FOV VCG-01112. We identified six putative SIX genes (FOXG_04863, FOXG_05755, FOXG_11033, SIX6, FOXG_02829 and FOXG_04805) through sequence homology searches. The most significant hits were found in both vegetative compatibility groups, with multiple high-scoring sequence alignments suggesting strong conservation of these effector proteins. This comparison noted that apart from SIX6, no other lineage-specific SIX was identified in the Australian genomes since all other SIX genes are non-lineage-specific SIX. Interestingly, we observed two genes with high sequence similarity to SIX6 in FOV VCG-01111 and FOV VCG-01112 (Table S6).

As noted earlier, we observed 121 singletons or unassigned genes for VCG-01111 and 132 for VCG-01112 and a few hundred genes specific to both lineages. Thus, there is potential that some of the species-specific genes are also potential signal peptides that contribute to the pathogenesis of our two genome assemblies. As such, we predicted signal peptides in all our protein sequences. We identified 1228 genes from the FOV VCG-01111 genome assembly, while FOV VCG-01112 had 1265 genes classified as signal peptides. After placing the signal peptides, we double-checked that the ortholog signal peptides identified earlier were also identified as signal peptides in our analysis. Table S7 shows that our analysis classified all the SIX genes orthologs as signal peptides. Given this validation, we looked at the potential for the unassigned genes to be putative lineage-specific single peptides and found that out of the 121 unassigned genes for FOV VCG-01111, 5 (4.12%) were putative signal peptides. Meanwhile, FOV VCG-01112 had only 3 (2.27%) of the 132 unassigned genes as putative signal peptides (Table S8). Thus, the remaining species-specific unassigned ortholog genes may be involved in functions contributing to these isolates’ pathogenicity and adaptation.

## 4. Discussion

In this study, we have constructed two highly contiguous and annotated genomes for the Australian *Fov* biotypes VCG-01111 and VCG-01112. These genomes were assembled using a third-generation sequencing approach that incorporates long reads (PacBio HiFi) and 3D chromosome structural information (HiC) resulting in two complete genome assemblies. These two new genomes join the existing list of 12 *Fov* genomes currently available at NCBI [47] (as of February 2025), providing a valuable addition to the *Fov* genome resources.

Our phylogenetic analyses reveal a significant evolutionary distance between the Australian biotypes of *Fov* and other global biotypes, corroborating previous findings that link VCG-01111 and VCG-01112 more closely to native Australian wild *Fusarium* species than to other *Fov* races [9]. These results reinforce the hypothesis that VCG-01111 and VCG-01112 are native to Australia and may have undergone a host switch following the introduction of cultivated cotton. Supporting this idea, the initial identification of VCG-01111 occurred in cotton fields surrounded by native Australian *Gossypium* species [2]. Our estimates suggest that the evolutionary divergence between Australian *Fov* strains and other *Fov* occurred approximately 3.4 MYA (Figure 2B), predating the polyploidization of cultivated cotton (1–2 MYA) but following the divergence of native Australian *Gossypium* (5–10 MYA) [48].

A key phenotypic distinction between Australian *Fov* strains and most other *Fov* strains is the absence of nematode interaction as a prerequisite for pathogenic infection of cotton [49]. While the devastating Race 4 strain is also capable of infecting cotton in the absence of nematodes, Australian biotypes have not been detected outside of Australia, suggesting an independent evolutionary origin of this trait. Given that Race 4 is closely related to Race 7 and Race 1 [20,21] and has undergone a more recent evolutionary split (Figure 2B), future studies should investigate the underlying molecular mechanisms that enable *Fov* strains, including Race 4, VCG-01111 and VCG-01112, to infect cotton without nematode interaction.

Our comparative genomic analyses did not reveal significant clustering between the Australian biotypes and Race 4 at the gene level. While both Australian biotypes share portions of the accessory chromosomes, they do not share all lineage-specific elements. Given that accessory chromosomes are known to play a role in Fusarium pathogenicity and host adaptation, it is expected that genetic differences influencing pathogenicity would reside within these regions. However, we did not observe a significant enrichment of signal peptides or gene numbers relative to chromosome size in the accessory chromosomes, contrasting with findings from the Race 7 *Fov* genome, where accessory chromosomes harbour genes contributing to pathogenicity [20]. Nonetheless, gene families associated with pathogenicity are enriched in the Australian genomes, and accessory chromosomes do contain other signal peptides that could contribute to adaptation and virulence.

Notably, genes unique to both Australian isolates are associated with extensive transcriptional regulation, secretory pathways, complex polysaccharide metabolism, and metabolite transport systems. This suggests that the Australian genomes have evolved enhanced transcriptional control and diverse metabolic capabilities, particularly in carbohydrate utilization and sophisticated transport mechanisms. These traits, combined with the enrichment of pathogenesis-related genes and hydrolytic enzymes, indicate the potential adaptation of the Australian biotypes for cotton infection. This is particularly relevant given the regional specificity of at least one of these genomes [9,50,51] and observed field phenotypic variations from standard *Fov* disease presentations [8]. However, genetic marker analyses suggest that these differences are unlikely to be due to the emergence of new or undetected isolates [7,52]. The observed genomic complexity supports the notion that variations in disease symptoms may arise from differential activation of intricate cellular pathways within the pathogen.

We identified orthologs of secreted-in-xylem (SIX) proteins, which are crucial for fungal pathogenicity, in the Australian biotype genomes through BLAST (v 2.16.0) analysis. However, this only included the six putative SIX genes, including SIX6, a known ortholog found in the Australian biotypes, which differentiated them from other *Fov* isolates lacking this gene [51]. The other five orthologs identified are not considered lineage-specific proteins for *Fol*; however, they do appear to be differentially expressed across different stages of infection development, as well as act as novel candidate effectors genes that cause cell death. [53]. The initial characterisation of these effector genes gives insight into their potential mechanism orchestrated by the pathogen during early infection stages. For instance, FOXG_0405 in *Fol* is significantly upregulated during the first two days post-inoculation. In comparison, FOXG_11033 is predominantly upregulated within four days post-inoculation, and the remaining four effector genes (FOXG_02829, FOXG_04863, FOXG_05755 and SIX6) were most upregulated at six days post-infection [53]. This orchestration of gene effectors at the early stages of infection could point towards distinct effects these proteins have in the plant cells required for successful invasion. Thus, the presence of these effectors in the Australian biotype genomes could indicate the likely molecular mechanism implemented by these pathogens to infect the cotton plants. To our knowledge, this is the first time these proteins have been identified within the Australian biotypes of *Fov*, which highlights the value of whole genome sequencing, annotation and evolutionary characterisation of pathogenic species.

While these SIX orthologs are likely contributors to pathogenicity, they may not be the sole determinants. We identified 121 genes without homologs, some of which encode predicted signal peptides. These findings open new avenues for proteomics and functional genomic studies to elucidate the infection mechanisms employed by Australian biotypes, which may differ from those of other *Fov* isolates.

Given the high similarity in host range and virulence among Australian biotypes [2], genetic differences beyond pathogenicity are likely associated with adaptation to environmental conditions. VCG-01111 is widely distributed, while VCG-01112 remains geographically restricted to its initial discovery region. One theory for this difference is that VCG-01111 was inadvertently spread via large earth-moving equipment that was situated in the Darling Downs where it was discovered. The *“Come Clean Go Clean”* procedures initiated after the discovery of *Fov* then restricted the further spread of *Fov*, preventing the movement of VCG-01112. However, considering the time elapsed since the initial discovery of *Fov* in Australia and the extensive cultivation of cotton, it is plausible that VCG-01111 possesses greater environmental adaptability than VCG-01112. This is corroborated by recent field data showing that cotton fields in Boggabilla, New South Wales, were traditionally infected by VCG-011112 and are now dominated by VCG-01111, indicating a complete displacement of VCG-01112 (*Linda J. Smith per personal communication*). The differences between the VCG-01111 and VCG-01112 genomes do not suggest a facile answer to their current distribution, but the lower gene count in VCG-01111 may contribute to its broader adaptability, while VCG-01112 may be more suited to a specialized ecological niche. Gene family evolution analyses indicate a complex and dynamic evolutionary trajectory, with notable genomic differences between the two Australian isolates despite their close genetic relationship. These differences may be driven by distinct ecological niches and structural genome variations [2,54].

We also observed that many functionally enriched genes involved cellular processes related to enhanced xenobiotic resistance and stress tolerance, supported by diverse metabolic capabilities and sophisticated transport systems. The combination of enriched drug response genes, stress signalling pathways and various catabolic processes indicates the evolution of robust survival mechanisms, potentially contributing to environmental resilience and adaptation in the Australian *Fov* isolates. These patterns of gene family evolution suggest multiple instances of independent adaption, and given the presence of other cotton species in Australia, this pathogen may have continued to acquire and lose functions that aid with host specificity, virulence and metabolic capabilities.

## 5. Conclusions

In this study, we have developed two new complete genome assemblies for the Australian Fov biotypes VCG-01111 and VCG-01112. Through comparative genomic analysis, we identified key genetic and functional differences that distinguish these biotypes from other *Fov* strains. For instance, we identify that four of the 15 chromosomes represent accessory chromosomes. We also noted that these accessory chromosomes had lower levels of gene diversity. We also identified potential novel signal peptides that can help explain the fungal pathogenicity. All in all, these new genome assemblies provide a valuable resource for understanding *Fov* pathogenicity, resistance mechanisms and the evolutionary processes that shaped these unique Australian biotypes.

## Data Availability

Raw reads with adapters removed and genome assemblies with basic gene annotation are available at NCBI under accessions SAMN45866337 and SAMN45866338. Genome assemblies with annotations are deposited under the BioProject PRJNA1199160, accession numbers JBMJCH000000000 and JBMJCI000000000 for VCG-01111 and VCG-01112, respectively. The genome assemblies can also be found in GenBank under GenBank accession ids GCA_049307005.1 and GCA_049306905.1 for both VCG-01111 and VCG-01112, respectively. We have also deposited the genomes and the annotation on the CSIRO Data Access Portal (accessed on 16 January 2025) [58] with permalink: https://doi.org/10.25919/13pj-dn18.

## Supplementary Materials

The following supporting information can be downloaded at: https://www.mdpi.com/article/10.3390/jof11070481/s1, Table S1: Genome assemblies used for genomic comparisons and phylogenetic analysis. Table S2: Gene Orthology Enrichment Results for the 442 Australian isolates unique gene clusters; Table S3: Gene Orthology Enrichment Results VCG-01111 gene family expansion and contraction; Table S4: Gene Orthology Enrichment Results VCG-01112 gene family expansion and contraction; Table S5: Gene Orthology Enrichment Results for the common ancestor of VCG-01111 and VCG-01112 gene family expansion and contraction; Table S6: Results from reciprocal best hit analysis of both VCG-01111 and VCG-01112 genes against the SIX gene from *Fov* race 7; Table S7: Signal peptide classification for all SIX genes orthologs in the VCG-01111 and VCG-01112 genome; Table S8: Novel putative signal peptides identified for each of the genomes. Figure S1. Raw HiC contact matrix for FOV-01111 used to complete the whole genome assembly. Figure S2. Raw HiC contact matrix for FOV-01112 used to complete the whole genome assembly. Figure S3. Location of telomere elements in the whole genome assembly VCG-01112. The blue triangles indicate the locations of telomeres on some chromosomes. Figure S4. Alignment comparison between the four whole genomes with FOV VCG-01111 denoting the chromosome to which it aligned as well as the strand orientation. Figure S5. Alignment comparison between the four whole genomes with FOV VCG-01112 denoting the chromosome to which it aligned as well as the strand orientation. Figure S6. Statistical comparison on the gene density of core chromosomes vs the Lineage Specific (accessory chromosomes) in the two genome assemblies. Although there is a clear signature for core chromosomes to contain more genes, the accessory chromosomes also showed a wide range of gene density. References [20,22,55,56,57] are cited in the Supplementary Materials.
