# Supplementary material for: Comparative Genomics Reveals Ancient and Unique Pathogenicity Features in Australian Fusarium oxysporum f. sp. vasinfectum
Source: J Fungi (Basel). 2025 Jun 25;11(7):481. doi: 10.3390/jof11070481 (PMC12299785; doi:10.3390/jof11070481)
Supplement: Supplementary file 1 [file jof-11-00481-s001.zip › jof-3668307-supplementary.pdf]

Supplementary Figures:

Comparative genomics reveals ancient and unique pathogenicity features in Australian *Fusarium oxysporum* f. sp. *Vasinfectum*

Angel David Popa-Baez[1]*, Linda J. Smith[2], Warwick N. Stiller[3], Melanie Soliveres[1], Gunjan Pandey[4], Christopher A. Saski[5], Don C. Jones[6] and Iain W. Wilson[1]

[1] CSIRO Agriculture and Food, Canberra, ACT 2601, Australia
[2] Ecosciences Precinct, Department of Primary Industries, Dutton Park, QLD 4102, Australia
[3] CSIRO Agriculture and Food, Locked Bag 59, Narrabri, NSW 2390, Australia
[4] CSIRO Environment, Canberra, ACT 2601, Australia
[5] Department of Plant and Environmental Sciences, Clemson University, Clemson, South Carolina, USA
[6] Cotton Incorporated, Cary, North Carolina, USA
* Correspondence: angel.popa@csiro.com;

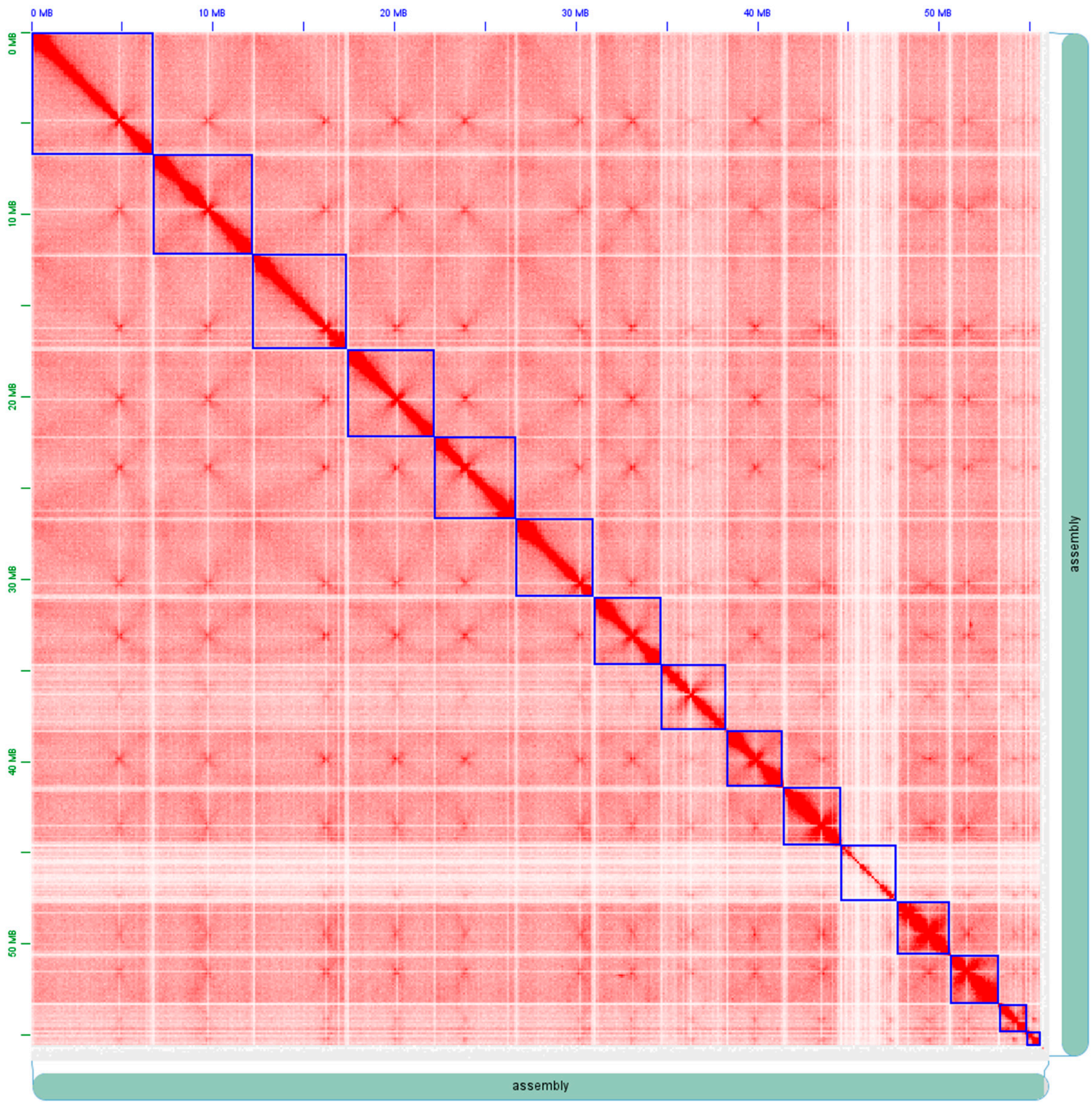

Figure S1. Raw HiC contact matrix for FOV-01111 used to complete the whole genome assembly

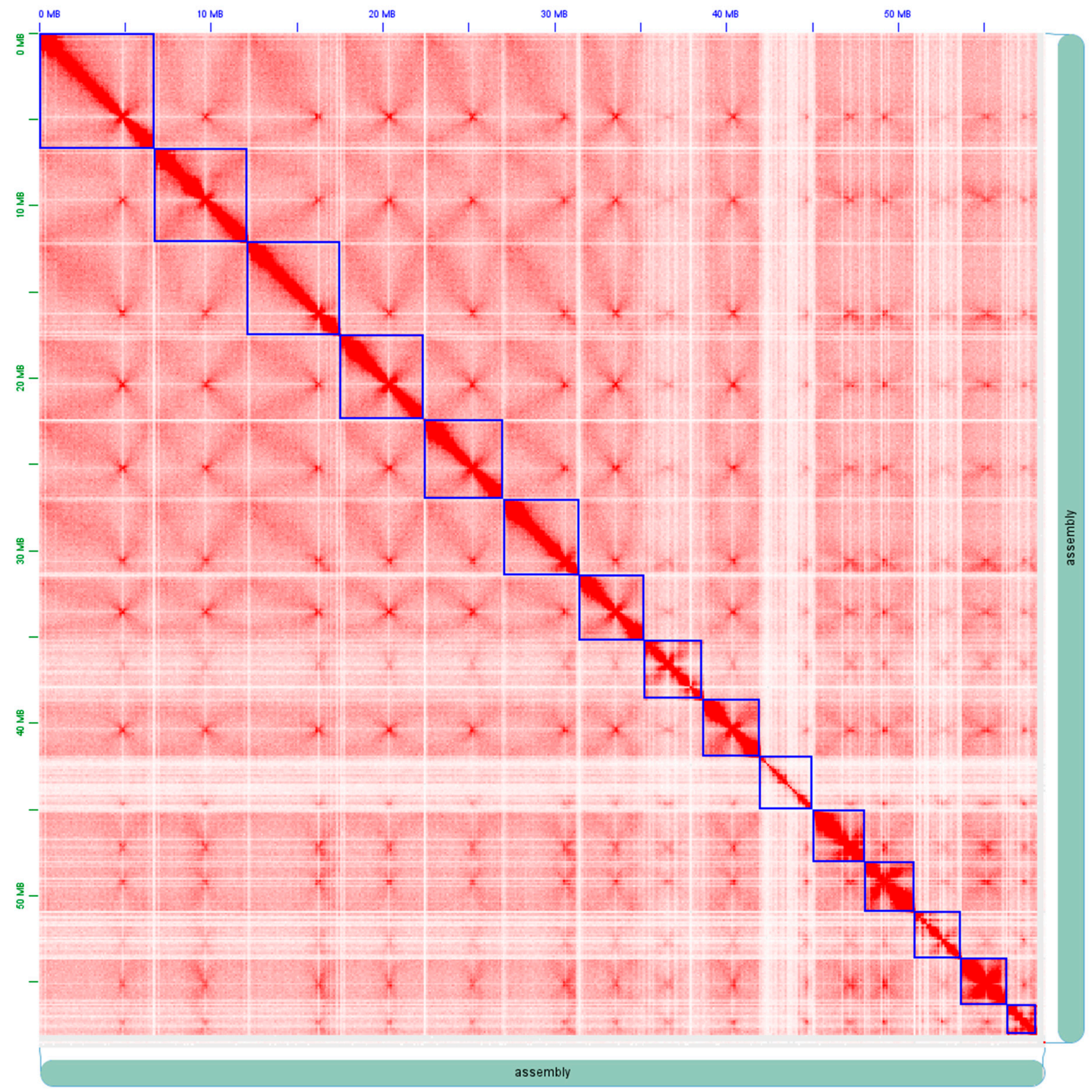

Figure S2. Raw HiC contact matrix for FOV-01112 used to complete the whole genome assembly

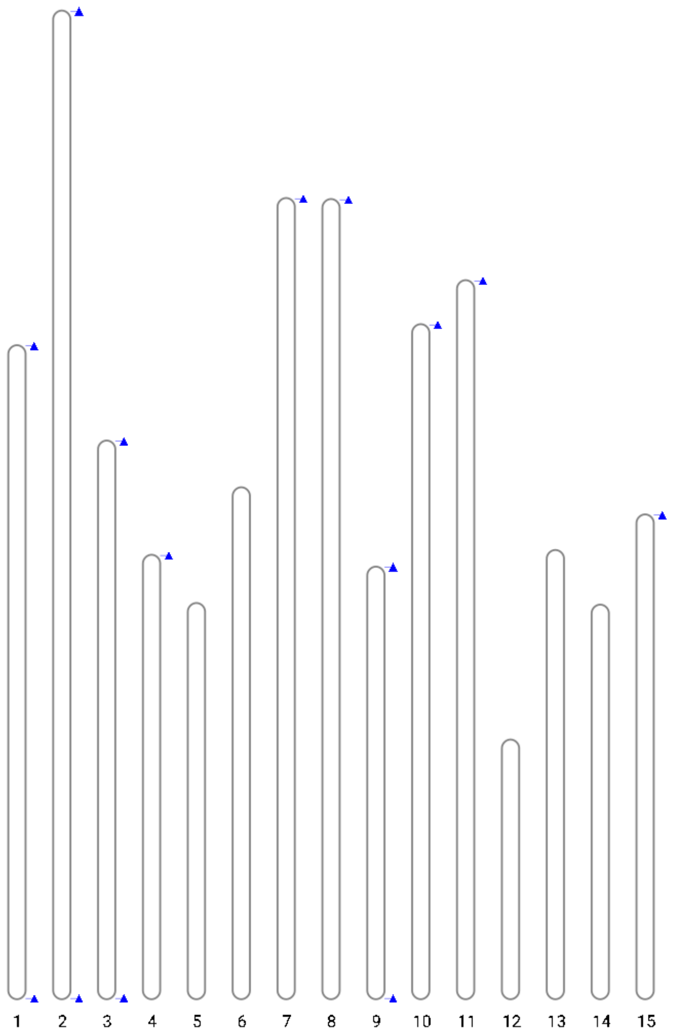

Figure S3. Location of telomere elements in the whole genome assembly VCG-01112. The blue triangles indicate the locations of telomeres on some chromosomes.

F11_Fov-1		
F11	Fov-1	Strand
CHR1	CP130297.1	R
CHR2	CP130301.1	R
CHR3		
CHR4	CP130298.1	R
CHR5	CP130299.1	
CHR6		
CHR7	CP130300.1	
CHR8	CP130302.1	
CHR9	CP130303.1	
CHR10	CP130304.1	R
CHR11	CP130305.1	
CHR12	CP130306.1	
CHR13	CP130307.1	R
CHR14		
CHR15		

F11_Fov-4		
Query	Target	Strand
CHR1	JAVSMJ010000001.1	R
CHR2	JAVSMJ010000005.1	R
CHR3		
CHR4	JAVSMJ010000004.1	R
CHR5	JAVSMJ010000003.1	R
CHR6		
CHR7	JAVSMJ010000002.1	R
CHR8	JAVSMJ010000007.1	R
CHR9	JAVSMJ010000008.1	R
CHR10	JAVSMJ010000009.1	
CHR11	JAVSMJ010000006.1	
CHR12	JAVSMJ010000011.1	
CHR13	JAVSMJ010000012.1	
CHR14		
CHR15		

F11_Fov-7		
Query	Target	Strand
CHR1	JBBJCF010000001.1	
CHR2	JBBJCF010000006.1	R
CHR3		
CHR4	JBBJCF010000002.1	R
CHR5	JBBJCF010000003.1	
CHR6		
CHR7	JBBJCF010000005.1	
CHR8	JBBJCF010000007.1	
CHR9	JBBJCF010000009.1	R
CHR10	JBBJCF010000010.1	R
CHR11	JBBJCF010000011.1	R
CHR12	JBBJCF010000004.1	
CHR13	JBBJCF010000013.1	R
CHR14		
CHR15		

F11_Fol		
Query	Target	Strand
CHR1	NC_030986.1	R
CHR2	NC_030987.1	
CHR3		
CHR4	NC_030989.1	R
CHR5	NC_030990.1	R
CHR6		
CHR7	NC_030992.1	R
CHR8	NC_030993.1	
CHR9	NC_030994.1	
CHR10	NC_030995.1	R
CHR11	NC_030996.1	R
CHR12	NC_030997.1	
CHR13	NC_030998.1	R
CHR14		
CHR15		

Figure S4. Alignment comparison between the four whole genomes with FOV VCG-01111 denoting the chromosome to which it aligned as well as the strand orientation.

F12_Fov-1		
Query	Target	Strand
CHR1	CP130297.1	R
CHR2	CP130301.1	R
CHR3		
CHR4	CP130298.1	R
CHR5	CP130299.1	
CHR6		
CHR7	CP130300.1	R
CHR8	CP130302.1	
CHR9	CP130303.1	
CHR10	CP130304.1	
CHR11	CP130305.1	
CHR12	CP130306.1	
CHR13	CP130307.1	R
CHR14		
CHR15		

F12_Fov-4		
Query	Target	Strand
CHR1	JAVSMJ010000001.1	R
CHR2	JAVSMJ010000005.1	R
CHR3		
CHR4	JAVSMJ010000004.1	R
CHR5	JAVSMJ010000003.1	R
CHR6		
CHR7	JAVSMJ010000002.1	
CHR8	JAVSMJ010000007.1	R
CHR9	JAVSMJ010000008.1	R
CHR10	JAVSMJ010000009.1	R
CHR11	JAVSMJ010000006.1	
CHR12	JAVSMJ010000011.1	
CHR13	JAVSMJ010000012.1	R
CHR14		
CHR15		

F12_Fov-7		
Query	Target	Strand
CHR1	JBBJCF010000001.1	
CHR2	JBBJCF010000006.1	R
CHR3		
CHR4	JBBJCF010000002.1	R
CHR5	JBBJCF010000003.1	
CHR6		
CHR7	JBBJCF010000005.1	R
CHR8	JBBJCF010000007.1	
CHR9	JBBJCF010000009.1	R
CHR10	JBBJCF010000010.1	
CHR11	JBBJCF010000011.1	R
CHR12	JBBJCF010000004.1	
CHR13	JBBJCF010000013.1	R
CHR14		
CHR15		

F12_Fol		
Query	Target	Strand
CHR1	NC_030986.1	R
CHR2	NC_030987.1	
CHR3		
CHR4	NC_030989.1	R
CHR5	NC_030990.1	R
CHR6		
CHR7	NC_030992.1	
CHR8	NC_030993.1	
CHR9	NC_030994.1	
CHR10	NC_030995.1	
CHR11	NC_030996.1	R
CHR12	NC_030997.1	
CHR13	NC_030998.1	R
CHR14		
CHR15		

Figure S5. Alignment comparison between the four whole genomes with FOV VCG-01112 denoting the chromosome to which it aligned as well as the strand orientation.

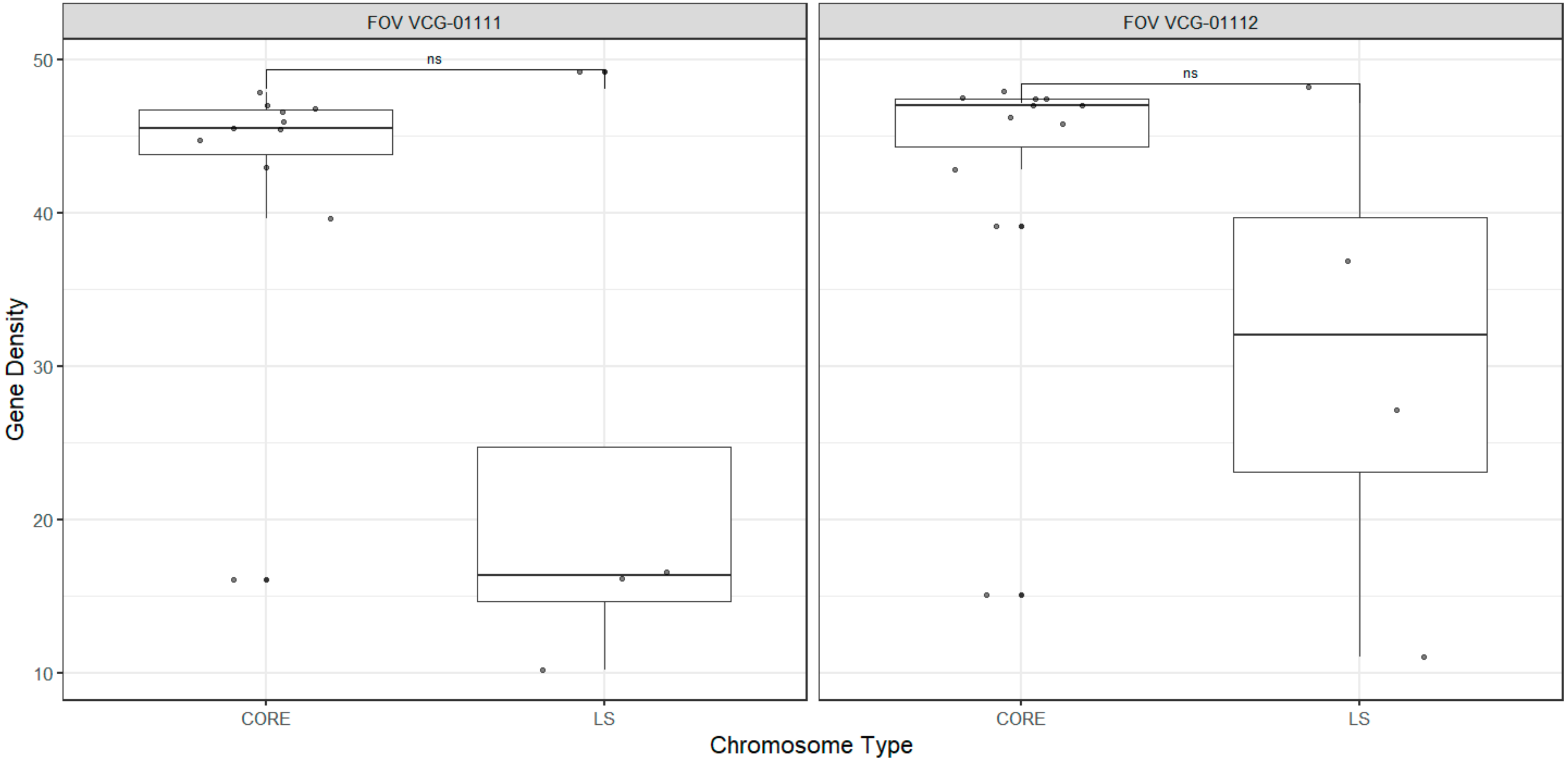

Figure S6. Statistical comparison on the gene density of core chromosomes vs the Lineage Specific (accessory chromosomes) in the two genome assemblies. Althtough there is a clear signature for core chromosomes to contain more genes, the accessory chromosomes also showed a wide range of gene density.
